# Supplementation of Trimethylamine N-Oxide or Betaine in Semen Improves Quality of Boar Spermatozoa Stored at 17 °C Following Hydrostatic Pressure Stress

**DOI:** 10.3390/life15101606

**Published:** 2025-10-15

**Authors:** Cheng Qin, Guangyuan Lu, Xiao Lin, Zhongkai Wang, Shiyu Yang, Liqiong Teng, Xin Lin, Fangfang Li, Shouping Huang, Chuanhuo Hu

**Affiliations:** 1 Guangxi Key Laboratory of Animal Breeding and Disease Control, College of Animal Science and Technology, Guangxi University, Nanning 530004, China; qq65518542@163.com (C.Q.);; 2 Agricultural Service Center of Nashe Township, Bama County, Hechi 547514, China; bmnsscxm@163.com; 3 Nanning Zhuangbo Biotechnology Co., Ltd., Nanning 530006, China; linxiao.vet@163.com (X.L.);; 4 Guangxi Livestock and Poultry Breeding Improvement Station, Nanning 530001, China; 5 Livestock Management Station, Bureau of Agriculture and Rural Affairs of Bama County, Hechi 547500, China

**Keywords:** trimethylamine N-oxide, Betaine, boar semen, 17 °C, hydrostatic pressure

## Abstract

HP, as an isotropic physical stress, has been widely applied in cell biology and reproductive research to simulate the effects of environmental pressure on cellular functions. In this study, the elastic silicone membrane of a novel bionic insemination catheter was employed as the pressure medium, with semen perfused into a sealed silicone chamber. As the silicone membrane underwent controlled deformation, the liquid inside the chamber generated a nearly uniform isotropic pressure, thereby maintaining spermatozoa in a stable HP environment. Boar sperm are susceptible to physiological and functional damage under HP stress, which can impair fertilization capacity. This study aimed to investigate the effects of TMAO, BET, or their combination on the quality of semen from eight Landrace boars under HP during storage at 17 °C (experiment repeated three times). Semen was collected using the manual collection method and treated with different concentrations of TMAO or BET. Sperm motility parameters were assessed using a CASA system to determine the optimal concentrations. Subsequently, experimental groups were established: the fresh group, HP control group, T group (optimal TMAO), B group (optimal BET), and H group (optimal TMAO + BET). The results showed that the optimal concentrations were 8 mmol/L for TMAO and 20 mmol/L for BET. Compared with the HP control group, the T, B, and H groups showed significantly improved sperm viability, mitochondrial membrane potential (MMP), and plasma membrane integrity (*p* < 0.05), and significantly reduced DFI, ROS, MDA, and NO contents (*p* < 0.05), while acrosome integrity showed no significant differences (*p* > 0.05). Additionally, the B group showed significantly increased T-AOC (*p* < 0.05). Non-targeted lipidomic analysis revealed 49 differential lipids in the T group, 262 in the B group, and 269 in the H group compared with the HP control. These differential lipids were mainly associated with PC, AcCa, and sphingolipid signaling pathways, with key sphingolipid pathway lipids including Cer, SM, and DG. These findings indicate that BET and TMAO + BET improve HP-induced sperm damage by modulating the sphingolipid signaling pathway and maintaining PC and AcCa levels, whereas TMAO alone may exert protective effects through additional mechanisms. In conclusion, TMAO, BET, or their combination effectively mitigates the detrimental effects of HP on boar sperm.

## 1. Introduction

During storage, spermatozoa are susceptible to damage from factors including temperature, osmotic pressure, hydrostatic pressure (HP), and oxidative stress (OS). Recently, a novel biomimetic insemination device (commercial name: KuaiPeiShu) was developed by Ningbo Sansheng Biotechnology Co., Ltd. (Ningbo, China), integrating an insemination catheter with a semen storage unit. However, when semen is preserved in its expandable silicone membrane, a certain degree of HP is generated, which may negatively affect sperm quality.

The cell membrane is among the most pressure-sensitive biological systems [1,2,3]. HP can affect protein folding, metabolic rate, and membrane stability, ultimately leading to cell lysis [4]. Membrane integrity is crucial for cellular function, including energy (ATP) production, ion and nutrient transport, and membrane-associated signal transduction. The physicochemical properties of membranes—such as fluidity, bending rigidity, lateral organization, and morphology—can modulate interactions between lipids and proteins, thereby regulating membrane protein function. These properties and functions are significantly influenced by osmotic pressure and HP [5,6,7,8,9]. Boar sperm membranes are rich in polyunsaturated fatty acids and are particularly vulnerable to reactive oxygen species (ROS), which can trigger lipid peroxidation chain reactions [10]. Lipids are fundamental structural components of cell membranes that are critical in energy storage, signal transduction, and the regulation of cellular homeostasis. Lipidomics allows us to systematically analyze cellular lipid composition and dynamic changes, thereby enhancing our understanding of cellular function, disease mechanisms, and metabolic regulation. Lipidomics is particularly valuable for elucidating the roles of lipids in physiological and pathological processes, as maintenance of lipid homeostasis is essential for cellular health. In spermatozoa, survival, maturation, and fertilization are significantly influenced by lipid homeostasis. Phospholipids and other lipid species are key components of the plasma membrane, regulating membrane fluidity, ion channel activity, and membrane-associated enzyme functions. Capacitation and the acrosome reaction, which are critical pre-fertilization processes, involve substantial lipid remodeling. Lipidomic analyses can therefore provide in-depth insights into these lipid changes [11]. Prolonged exposure of boar sperm to HP stress may induce OS, ultimately impairing sperm physiological functions.

Trimethylamine N-oxide (TMAO) is a small-molecule osmoprotectant widely present in marine organisms, helping them withstand the high HP of deep-sea environments [12,13,14]. TMAO can counteract high HP by modulating membrane lipid composition to maintain membrane fluidity, reducing ROS generation, mitigating lipid peroxidation and OS damage, and enhancing cell viability [15]. Betaine (BET) is a quaternary ammonium compound abundantly found in sugar beet, spinach, whole grains, and seafood. Its primary functions include regulating cellular osmotic pressure to minimize the impact of environmental changes and lowering ROS levels to reduce oxidative damage [16].

Currently, studies on the effects of HP on the quality of boar semen during room-temperature storage are limited. The influence of TMAO and BET on spermatozoa was investigated in this study by assessing semen motility parameters, antioxidant capacity, resistance to HP stress, and artificial insemination outcomes, providing theoretical support for their application in enhancing HP tolerance and room-temperature preservation of boar sperm.

## 2. Materials and Methods

### 2.1. Chemicals

TMAO (317594-5G, Sigma, St. Louis, MO, USA) and BET (B2629, Sigma, USA) were purchased.

### 2.2. Experimental Animals and Sources

Semen was collected from eight Landrace boars with similar body condition and aged between 24 and 48 months at the boar station of Guangxi Zhuangbo Biotechnology Co., Ltd. (Nanning, China). The semen samples used in this study were collected from March to July, during which the boars were collected at a frequency of 2–3 times per week to ensure stable semen quality. Ultimately, semen samples from six collections were selected as experimental materials. All feeding and management practices followed the company’s standard protocols for breeding boars. As the sample collection spanned several months, seasonal factors might have caused variations in semen quality. Therefore, in this experiment, each semen sample underwent a preliminary quality assessment (volume, concentration, sperm motility, etc.), and only those meeting the predefined quality standards were included in subsequent experiments; samples that did not meet the standards were recorded and excluded.

### 2.3. Experimental Designs

The study included a fresh semen group, a pressurized control group, and groups treated with different concentrations of TMAO or BET. Except for the fresh group, in which boar semen was stored in standard storage bottles, all other groups were preserved in the novel bionic insemination catheter (HP: 105 kPa). Kinematic parameters were recorded at 0 h, 48 h, 96 h, and 144 h after treatment. The total motility (TM), curvilinear velocity (VCL), average path velocity (VAP), and straight-line velocity (VSL) were assessed. The optimal concentrations of TMAO and BET were identified based on these parameters and used for subsequent experiments.

Experimental groups included the fresh control (freshly collected semen, diluted and stored in conventional semen tubes), HP control (semen stored in the novel biomimetic insemination device for 96 h), T group (semen supplemented with TMAO and stored in the device for 96 h), B group (semen supplemented with BET and stored in the device for 96 h), and H group (semen supplemented with TMAO + BET and stored in the device for 96 h). Mitochondrial membrane potential (MMP), DNA fragmentation index (DFI), plasma membrane integrity, acrosome integrity, ROS levels, malondialdehyde (MDA) content, nitric oxide (NO) content, and total antioxidative capacity (T-AOC) were assessed, and each experiment was performed in triplicate. Changes in lipid metabolism in boar sperm following hydrostatic pressure stress were investigated with untargeted lipidomics, with each experiment repeated six times.

### 2.4. Semen Collection and Dilution

Semen collection method: The boar was guided onto a dummy sow, and the collector wore double-layer gloves before starting the procedure. Once the boar mounted the dummy and maintained a stable position, preputial urine was expelled, and the area was disinfected. The prepuce and surrounding region were wiped with a potassium permanganate solution and then rinsed with clean water. After removing the outer gloves, the boar’s penis was grasped to perform semen collection. During the collection process, care was taken to minimize bacterial contamination, and the collection cup was maintained at 37–39 °C. After collection, the semen was promptly diluted.

According to the instructions, the diluent was pre-mixed with sterile distilled water, thoroughly mixed, and placed in a water bath at 37 °C for later use. The temperature of the fresh semen was measured, and the diluent was adjusted to match this temperature. Fresh semen was first diluted 1:1 (*v*/*v*) with the diluent, and after standing for 3–5 min, 20 μL of semen was placed on a microscope slide for counting under a 20× objective. Based on the results of cell counting, semen was further diluted to a density of 2 × 10^7^–5 × 10^7^ sperm/mL. Only samples with motility > 90% and abnormality rate < 5%, as determined by the CASA system, were considered. Samples were transferred to the laboratory within 12 h and stored at a constant 17 °C, with gentle mixing every 10 h.

### 2.5. Methods

#### 2.5.1. Assessment of Sperm Kinematic Parameters

Sperm kinematic parameters were evaluated using a computer-assisted sperm analysis system (CASA, CEROS II, IMV, Shanghai, China). A 6 μL semen sample was placed on a pre-warmed slide at 37 °C, covered with a coverslip, and mounted on a 37 °C heated stage. Observations were made under a 400× magnification microscope. Fields free of debris and air bubbles were selected, and images were captured once the liquid surface stabilized, with approximately 600 spermatozoa recorded per sample.

#### 2.5.2. Assessment of Sperm Viability

Sperm viability was evaluated using the Sperm Vitality Kit (Eosin–Nigrosin method, Cat: G2581, Solarbio, Beijing, China). Semen samples were centrifuged at 2000 r/min for 5 min, and the supernatant and impurities were discarded. The sperm pellet was resuspended in phosphate-buffered saline, followed by centrifugation (2000 r/min, 5 min) and removal of the supernatant. This washing procedure was repeated three times to obtain purified spermatozoa. Smears were prepared according to the manufacturer’s instructions. After the smears were air-dried, sperm viability was examined under a light microscope. Evaluation criteria were as follows: viable spermatozoa displayed unstained (white) heads, non-viable spermatozoa exhibited red-stained heads, and the background appeared dark purple. For each slide, ≥200 spermatozoa were counted, and the percentage of unstained (viable) spermatozoa relative to the total was calculated.

#### 2.5.3. Assessment of Sperm MMP

Following sperm washing, mitochondrial membrane potential was evaluated using the Mitochondrial Membrane Potential Assay Kit with JC-1 (C2006, Beyotime, Shanghai, China) according to the manufacturer’s instructions. A 10 μL sperm suspension was prepared as a smear, observed under a fluorescence microscope. The acquired fluorescence images were analyzed using ImageJ 1.54f software to determine the mean fluorescence intensity.

#### 2.5.4. Assessment of Sperm DFI

Following sperm washing, DNA fragmentation was evaluated using the Nuclear Fragmentation Detection Kit (Cellpro, Ningbo, Zhejiang, China) according to the manufacturer’s protocol. Fluorescence images were captured and recorded under a fluorescence microscope, and ImageJ software was used for image analysis. The DFI (%) was calculated using the following formula: DFI (%) = Mean red fluorescence intensity/(Mean (green + red) fluorescence intensity).

#### 2.5.5. Assessment of Sperm Plasma Membrane Integrity

Following sperm washing, plasma membrane integrity was evaluated using the hypo-osmotic swelling test. A fructose hypo-osmotic solution was pre-warmed at 37 °C in a water bath, and an appropriate volume of the solution was added to the sperm pellet to adjust the final sperm concentration to 2–5 × 10^6^/mL. The fructose hypo-osmotic solution–sperm mixture was then incubated at 37 °C for 30 min. Subsequently, a 10 μL aliquot was used to prepare smears, which were examined under a light microscope at 400× magnification. The proportion of spermatozoa with intact plasma membranes was calculated. Spermatozoa with coiled or curved tails were classified as having intact plasma membranes, whereas spermatozoa with straight tails or tails bent at an angle greater than 90° were considered to have disrupted membranes.

#### 2.5.6. Assessment of Sperm Acrosome Integrity

Following sperm washing, acrosome integrity was evaluated using the Coomassie Brilliant Blue staining method. The Coomassie Brilliant Blue staining jar was pre-warmed in a 37 °C water bath. The sperm pellet was resuspended in 1 mL of 3.7% paraformaldehyde and incubated at room temperature for 30 min, followed by centrifugation at 2000 r/min for 5 min to remove the supernatant. The pellet was washed once with Phosphate-buffered saline (PBS) (2000 r/min, 5 min) and resuspended in 600 μL PBS. A 10 μL aliquot was smeared on a slide. After being air-dried, the slides were immersed in the pre-warmed staining jar for 5 min. The slides were gently rinsed with ultrapure water before they were air-dried at room temperature and examined under a light microscope at 400× magnification for sperm counting.

#### 2.5.7. Assessment of ROS Levels in Boar Sperm

Following sperm washing, intracellular ROS levels were assessed using the ROS Assay Kit (DCFH-DA, Beyotime, Shanghai, China) according to the manufacturer’s instructions. A 10 μL sperm pellet was smeared on a slide, fluorescence images were captured under a fluorescence microscope, and the mean fluorescence intensity was quantified using Image J software.

#### 2.5.8. Assessment of T-AOC Activity, NO Content, and MDA Concentration in Boar Sperm

Following sperm washing, T-AOC, NO, and MDA levels were measured using the Total Antioxidant Capacity Assay Kit (ABTS method, A015-2-1, Nanjing Jiancheng Bioengineering Institute, Nanjing, China), NO Assay Kit (A013-2-1, Nanjing Jiancheng Bioengineering Institute, China), and Lipid Peroxidation MDA Assay Kit (S0131S, Beyotime, Shanghai, China), respectively, according to the manufacturers’ instructions. Optical density (OD) values were determined at the corresponding wavelengths for each assay. T-AOC activity, NO content, and MDA concentration were calculated based on the provided formulas or standard curves.

#### 2.5.9. Untargeted Lipidomics Analysis

##### Sample Preparation

Sperm samples were transferred into 2 mL centrifuge tubes containing a 6 mm grinding bead, and 280 μL of extraction solution (methanol/water = 2:5, *v*/*v*) and 400 μL of methyl tert-butyl ether (MTBE) were added. The samples were homogenized for 6 min (−10 °C, 50 Hz) using a grinder, followed by low-temperature ultrasonication for 30 min (5 °C, 40 kHz). The samples were then incubated at −20 °C for 30 min and centrifuged for 15 min (12,000 r/min, 4 °C). A 350 μL aliquot of the supernatant was transferred to a new Eppendorf tube and evaporated to dryness under nitrogen gas. The dried extract was reconstituted in 100 μL extraction solution (isopropanol/acetonitrile = 1:1, *v*/*v*), vortexed for 30 s, and subjected to low-temperature ultrasonication for 5 min (5 °C, 40 kHz). Finally, the samples were centrifuged for 10 min (12,000 r/min, 4 °C), and the supernatant was transferred into LC vials with inserts for subsequent analysis.

##### Quality Control

For each sperm sample, 20 μL of the supernatant was thoroughly mixed to prepare quality control (QC) samples. QC samples were generated by combining equal volumes of extracts from all individual samples. Each QC sample had the same volume as the experimental samples and was processed using the same procedure. During LC-MS analysis, one QC sample was injected after every 5–15 experimental samples to monitor the stability and reproducibility of the analytical process.

##### LC-MS Analysis

(1) Chromatographic Separation: Samples were separated using an Accucore C30 column (100 mm × 2.1 mm i.d., 2.6 μm; Thermo, Waltham, MA, USA). Mobile phase A consisted of 50% acetonitrile in water containing 0.1% formic acid and 10 mM ammonium acetate, and mobile phase B consisted of acetonitrile/isopropanol/water (10:88:2, *v*/*v*/*v*) containing 0.02% formic acid and 2 mM ammonium acetate. The injection volume was 5 μL, and the column temperature was maintained at 40 °C. The gradient program was as follows:
  • 0–4 min: A decreased from 65% to 40%, B increased from 35% to 60%;
  • 4–12 min: A decreased from 40% to 15%, B increased from 60% to 85%;
  • 12–15 min: A decreased from 15% to 0%, B increased from 85% to 100%;
  • 15–17 min: A held at 0%, B held at 100%;
  • 17–18 min: A increased from 0% to 65%, B decreased from 100% to 35%;
  • 18–20 min: A held at 65%, B held at 35%.
(2) Mass Spectrometry Detection: Samples were analyzed using electrospray ionization (ESI) in both positive and negative ion modes. The MS parameters were as follows: scanning range, 200–2000 *m*/*z*; sheath gas flow, 60 psi; auxiliary gas flow, 20 psi; ion source temperature, 370 °C; ionization voltage, +3000 V (positive mode) and −3000 V (negative mode); collision energy, 20–40–60%.

##### Detection of Diacylglycerol (DG), Phosphatidylcholine (PC), Sphingomyelin (SM), Ceramide (Cer), and Acylcarnitine (AcCa) Concentrations in Boar Sperm

Following the washing procedure described in Section 2.5.2, the supernatant of preserved sperm samples was discarded, and the pellet was resuspended in an appropriate volume of PBS. Sperm were then sonicated in an ice-water bath using a sonicator (3 s on, 7 s off, total 3 min) to achieve cell lysis. The samples were centrifuged at 3000 r/min for 20 min, and the supernatant was transferred to a new centrifuge tube and kept on ice until analysis. Concentrations of diacylglycerol (DG, MM-98440O1), phosphatidylcholine (PC, MM-78474O1), sphingomyelin (SM, MM-78039O1), ceramide (Cer, MM-78236O1), and acetyl-CoA (AcCa, MM-927206O1) were determined using ELISA kits (Meimian, Yancheng, Jiangsu, China) according to the manufacturers’ instructions. Optical density (OD) was measured at 450 nm using a full-wavelength microplate reader. The concentrations of DG, PC, SM, Cer, and AcCa in each sample were calculated based on the standard curve and the OD values of the sample wells.

##### Data Analysis

All experimental data were analyzed using one-way analysis of variance (ANOVA) in SPSS 26.0 software and are presented as mean ± standard deviation. Graphs were generated using GraphPad Prism 10 software. Differences were considered statistically significant at *p* < 0.05 and not significant at *p* > 0.05.

## 3. Results

### 3.1. Experiment I

#### 3.1.1. Hydrostatic Pressure Measurement of the Novel Biomimetic Insemination Device

A 60 mL semen sample was loaded into the novel biomimetic insemination device. An intelligent digital pressure gauge was inserted into the tail end of the device, and readings were recorded after stabilization for 3 s. The measurement was repeated five times, and the average HP was determined to be 105 kPa.

#### 3.1.2. Effects of TMAO or BET on Kinematic Parameters of Boar Sperm Under HP Stress During Room-Temperature Storage

**Figure 1:**
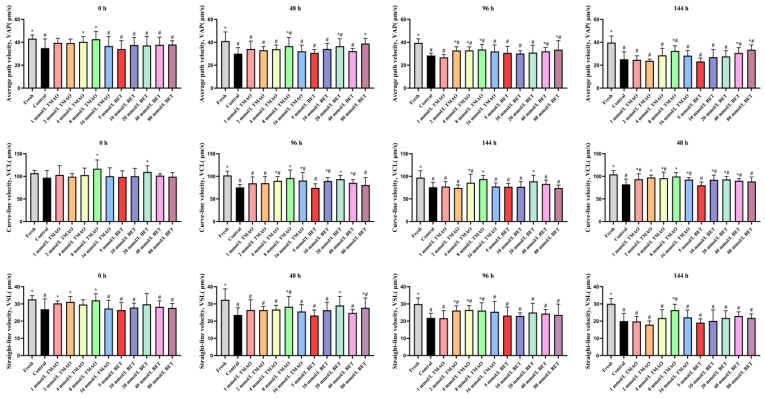
Effects of different concentrations of TMAO or BET on kinematic parameters of boar sperm under HP stress. Data are presented as mean ± SD (*n* = 3). Symbols indicate statistical significance: # compared with the fresh control group, * compared with the HP control group, *p* < 0.05; no symbol indicates *p* > 0.05.

As shown in Table 1 and Figure 1, semen loading into the novel biomimetic insemination device at 0 h resulted in a decrease in sperm quality. With increasing storage time, all kinematic parameters exhibited a time-dependent decline, with the HP control and 80 mmol/L BET groups showing the most pronounced reductions (Supplementary Table S1).

At 0 h of room-temperature storage after loading, TM in all experimental groups was significantly lower than in the fresh control (*p* < 0.05). Compared with the HP control, no significant differences in TM were observed among the experimental groups except for the fresh control (*p* > 0.05). The fresh control exhibited significantly higher VAP and VSL (*p* < 0.05). In the 8 mmol/L TMAO group, VCL, VAP, and VSL were significantly increased (*p* < 0.05). In the 20 mmol/L BET group, VCL was significantly elevated (*p* < 0.05).

At 48 h, all kinetic parameters (TM, VCL, VAP, and VSL) of the fresh group, the 8 mmol/L TMAO group, and the 20 mmol/L BET group were significantly higher than those of the pressurized control group (*p* < 0.05), with the greatest improvements observed in these groups.

At 96 h, among the different TMAO concentrations, the 8 mmol/L TMAO group showed the most pronounced increases in all kinetic parameters compared with the pressurized control group (*p* < 0.05); among the different BET concentrations, the 20 mmol/L BET group exhibited the greatest increases in all kinetic parameters (*p* < 0.05), whereas the 80 mmol/L BET group showed a significant decrease in TM (*p* < 0.05).

At 144 h, among the different TMAO concentrations, the 8 mmol/L TMAO group showed the most marked improvement in kinetic parameters compared with the pressurized control group (*p* < 0.05); among the different BET concentrations, the 20 mmol/L BET group showed the greatest improvement, with TM and VCL significantly increased (*p* < 0.05).

In practical applications of semen production, diluted boar semen is generally used within 96 h after loading. The 8 mmol/L TMAO group and the 20 mmol/L BET group exhibited the best improvements in kinematic parameters compared with the control. Therefore, for subsequent experiments, semen was stored for 96 h in the T group (8 mmol/L TMAO), B group (20 mmol/L BET), and H group (8 mmol/L TMAO + 20 mmol/L BET).

### 3.2. Experiment II: Effects of TMAO or BET on the Antioxidative Capacity of Boar Semen Under HP Stress

As shown in Table 2, the T group, B group, and H group showed significantly improved plasma membrane integrity, viability, and MMP levels and reduced DFI levels (*p* < 0.05). HP stress had no significant effect on acrosome integrity (*p* > 0.05, Supplementary Figures S1–S3).

As shown in Table 3, compared with the fresh control group, ROS levels were significantly increased in all other groups (*p* < 0.05), with the highest levels observed in the HP control group. Compared with the HP control group, ROS levels were significantly reduced in all treatment groups (*p* < 0.05). These results indicate that HP stress leads to excessive ROS accumulation in sperm, while supplementation with TMAO or BET effectively reduces ROS accumulation, although not to the level of the fresh control group (Supplementary Figures S4).

Compared with the fresh control group, MDA content was significantly increased in the HP control group (*p* < 0.05), while no significant differences were observed in the other treatment groups (*p* > 0.05). Compared with the HP control group, MDA content was significantly reduced in the fresh control group, T group, H group, and B group (*p* < 0.05).

Compared with the fresh control group, T-AOC levels were significantly reduced in the HP control group, T group, and H group *(p* < 0.05), while no significant difference was observed in the B group (*p* > 0.05). Compared with the HP control group, T-AOC levels were significantly increased in the fresh control group and B group (*p* < 0.05), with no significant differences in the other treatment groups (*p* > 0.05).

Compared with the fresh control group, NO content was significantly increased in the HP control group (*p* < 0.05), while no significant differences were observed in the other treatment groups (*p* > 0.05). Compared with the HP control group, NO content was significantly reduced in the fresh control group, T group, H group, and B group (*p* < 0.05).

Overall, these results demonstrate that supplementation with TMAO, BET, or their combination effectively reduced MDA and NO content in boar sperm under HP stress, while the B group was able to maintain T-AOC levels.

### 3.3. Experiment III: Effects of TMAO or BET on the Lipid Profile of Boar Sperm During Room-Temperature Storage

#### 3.3.1. PCA

**Figure 2:**
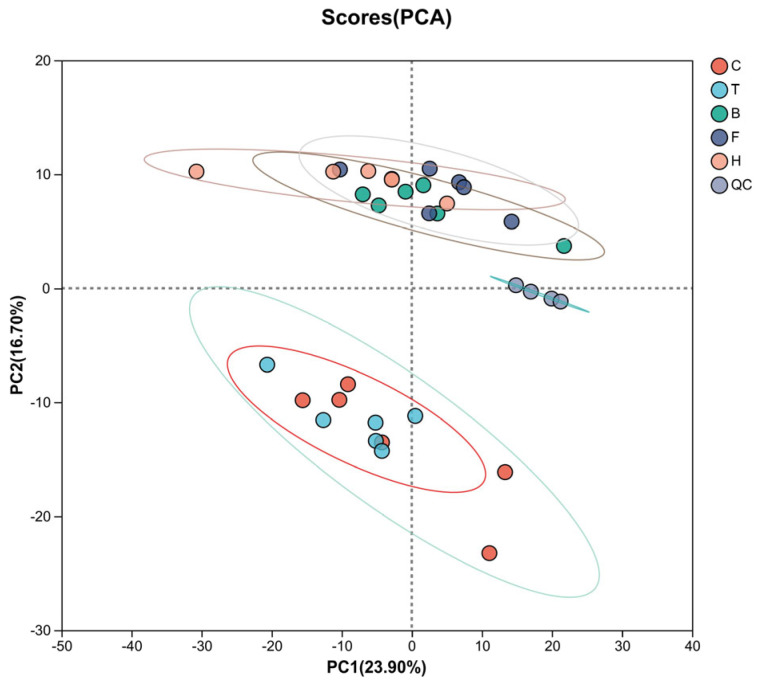
PCA score plot of boar sperm stored at room temperature under HP stress. C: pressurized control group; T: 105 kPa + TMAO group; B: 105 kPa + BET group; F: fresh sperm group; H: 105 kPa + TMAO + BET group; QC: quality control.

Principal component analysis (PCA) was performed for all groups. As shown in Figure 2, all groups exhibited clear separation except for the T group and C group, which showed some overlap. This indicates significant differences among the groups. Although T and C overlapped, subsequent analyses were not affected. QC samples clustered tightly, and all experimental samples fell within the 95% confidence interval, indicating good repeatability and reliability of the experiment.

#### 3.3.2. OPLS-DA Analysis

**Figure 3:**
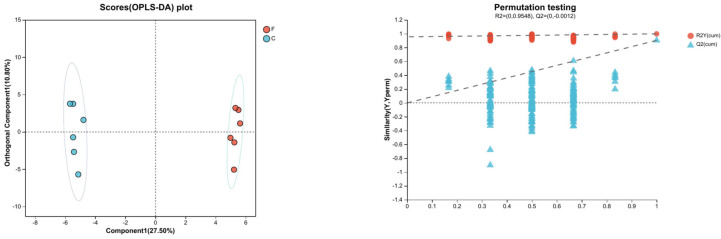
OPLS-DA score plot (**left**) and permutation test plot (**right**) of boar sperm stored at room temperature under HP stress. F: fresh sperm group; C: pressurized control group.

Orthogonal partial least squares discriminant analysis (OPLS-DA) was performed to identify and maximize differences in lipid metabolism among the experimental groups. Model fit and predictive ability were evaluated by R^2^ and Q^2^ values, where R^2^ > 0.9 and Q^2^ > 0.5 indicate no overfitting and good predictive ability. As shown in Figure 3, F vs. C, B vs. C, and H vs. C exhibited clear separation with R^2^ > 0.9 and Q^2^ > 0.5, whereas T vs. C showed R^2^ > 0.9 but Q^2^ < 0.5. These results indicate that the F vs. C, B vs. C, and H vs. C models were not overfitted and had good predictive ability, while the T vs. C model was not overfitted but had relatively poor predictive ability (Supplementary Figure S5).

#### 3.3.3. Differential Lipid VIP Analysis

**Figure 4:**
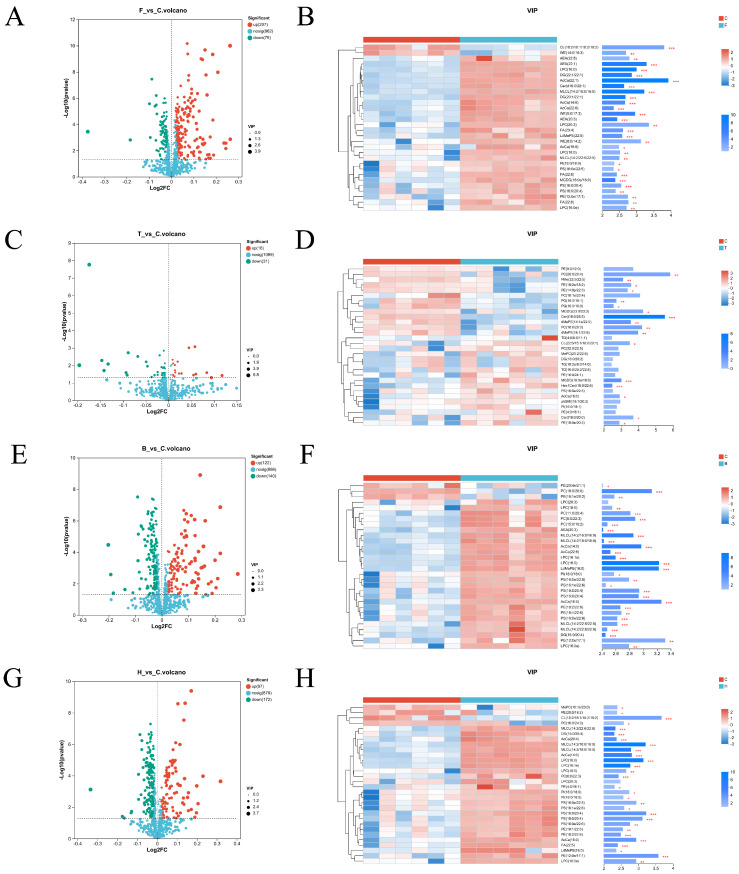
Volcano plot (**left**) and VIP analysis plot (**right**) of inter-group differences in boar sperm stored at room temperature under HP stress. F: fresh sperm group; C: HP control group; T: 105 kPa + TMAO group; B: 105 kPa + BET group; H: 105 kPa + TMAO + BET group. (**A**,**B**) F vs. C group; (**C**,**D**) T vs. C group; (**E**,**F**) B vs. C group; (**G**,**H**) H vs. C group.

Differential lipids between groups were analyzed using VIP values (VIP > 1) and *p*-values (*p* < 0.05). Volcano plots of differential lipids were generated based on fold change analysis and *t*-tests. Figure 4A,C,E,G show volcano plots of differentially expressed lipids, with red indicating upregulated lipids, green indicating downregulated lipids, and blue indicating non-significant lipids. In the F vs. C group, 286 differential lipids were identified, including 207 upregulated and 79 downregulated. In the T vs. C group, 49 differential lipids were identified, including 18 upregulated and 31 downregulated. In the B vs. C group, 262 differential lipids were identified, including 122 upregulated and 140 downregulated. In the H vs. C group, 269 differential lipids were identified, including 97 upregulated and 172 downregulated.

Figure 4B,D,F,H present VIP plots of differential lipids among the experimental groups, reflecting relative changes in lipid expression. Each row represents a lipid, with color gradients indicating relative expression levels and significance (* indicates *p* < 0.05, ** indicates *p* < 0.01, *** indicates *p* < 0.001). In the right panel, the length of the blue bars represents the magnitude of differences between groups, and the color intensity reflects statistical significance.

#### 3.3.4. KEGG Pathway Enrichment Analysis

**Figure 5:**
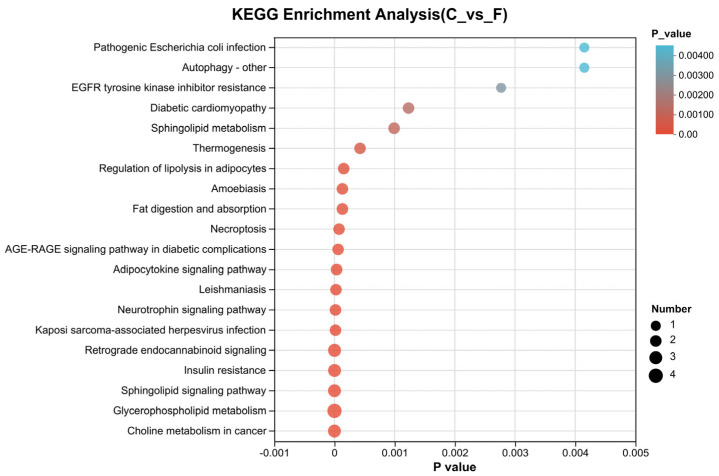
KEGG pathway enrichment analysis bubble plot of boar sperm stored at room temperature under HP stress. F: fresh sperm group; C: pressurized control group.

Differential lipids were subjected to KEGG pathway enrichment analysis to investigate the effects of TMAO, BET, or their combination on signaling pathways in boar sperm under HP stress. As shown in Figure 5, differential lipids in the F vs. C, T vs. C, B vs. C, and H vs. C comparisons were mainly enriched in glycerophospholipid metabolism, sphingolipid signaling pathway, choline metabolism in cancer, retrograde endocannabinoid signaling, insulin resistance, and adipocytokine signaling pathways. These results indicate that HP-induced damage affects lipid metabolic pathways in boar sperm, while treatment with TMAO or BET can modulate these pathways to mitigate HP-induced damage (Supplementary Figure S6).

#### 3.3.5. Screening of Differential Lipid Molecules and Signaling Pathways

**Figure 6:**
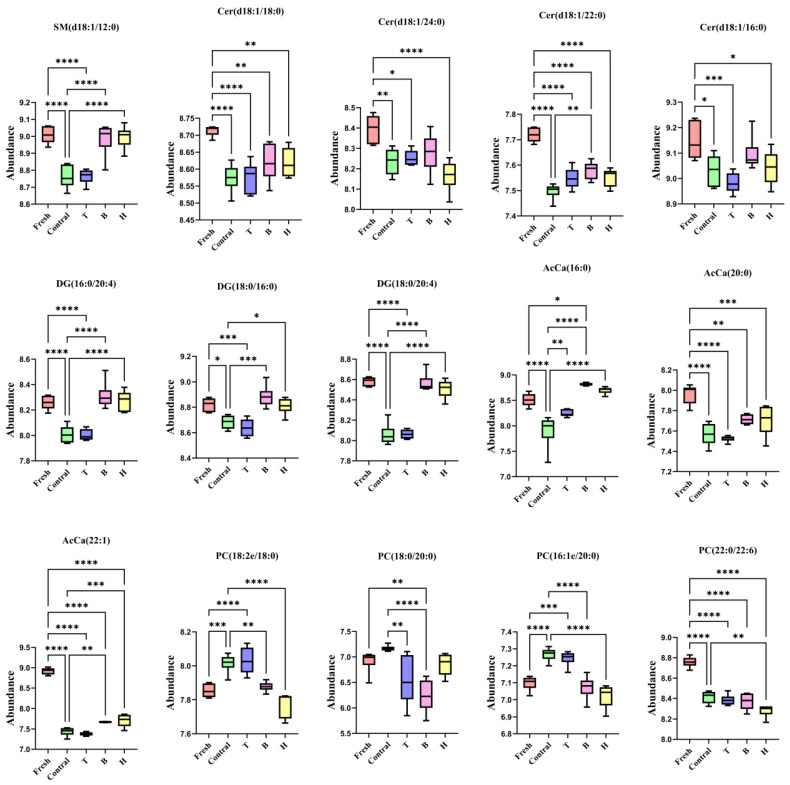
Expression levels of differential lipids in boar sperm stored at room temperature under HP stress. * indicates *p* < 0.05, ** indicates *p* < 0.01, *** indicates *p* < 0.001, and **** indicates *p* < 0.0001.

KEGG enrichment analysis of differential lipids revealed that the most consistently enriched and highly differential lipid metabolic pathways across F vs. C, T vs. C, B vs. C, and H vs. C were the sphingolipid signaling pathway and glycerophospholipid metabolism. Within the sphingolipid signaling pathway, the main differential lipids were SM, Cer, and DG, whereas in glycerophospholipid metabolism, the most prominent differential lipids were PC and AcCa. As shown in Figure 6, the expression levels of SM, Cer, DG, PC, and AcCa in the B and H groups were restored to levels close to the fresh group, whereas differences between the T and C groups were relatively small.

#### 3.3.6. Effects of TMAO or BET on DG, PC, SM, Cer, and AcCa Levels in Boar Sperm

**Figure 7:**
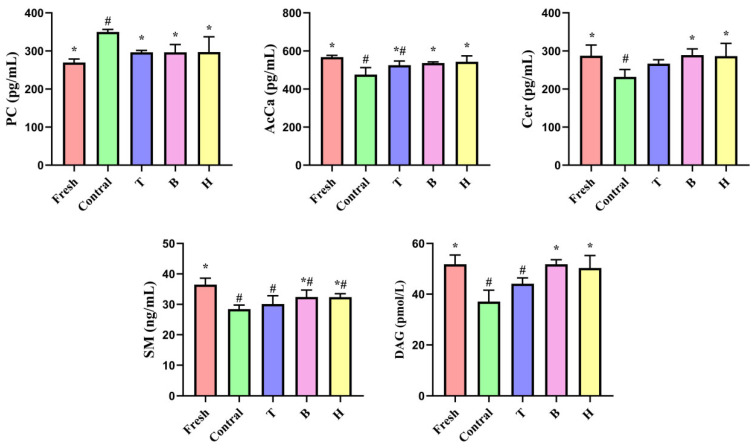
Effects of TMAO or BET on the contents of DG, PC, SM, Cer, and AcCa in boar sperm under HP stress during liquid storage. Data are presented as mean ± SD (*n* = 3). Symbols indicate statistical significance: # compared with the fresh control group, * compared with the HP control group, *p* < 0.05; no symbol indicates *p* > 0.05.

Lipidomic analysis indicates that HP stress affected SM, Cer, and DG in the sphingolipid signaling pathway, and significantly influenced PC and AcCa levels, which are closely related to sperm plasma membrane stability, fluidity, and antioxidative capacity. ELISA kits were used to quantify these five differential lipids and verify their trends. Compared with the fresh group, the HP control group exhibited significant decreases in Cer, DG, AcCa, and SM (*p* < 0.05), and a significant increase in PC (*p* < 0.05) (Figure 7). Supplementation with BET or TMAO + BET partially alleviated these changes, with Cer, DG, PC, and AcCa maintained at levels not significantly different from the fresh group (*p* > 0.05), whereas SM levels were partially improved but remained significantly lower than the fresh group (*p* < 0.05). In the TMAO group, SM, DG, and AcCa were significantly reduced (*p* < 0.05), while Cer and PC showed no significant differences (*p* > 0.05). These results indicate that the trends observed in the five differential lipid molecules are consistent with the lipidomic analysis, suggesting that supplementation with TMAO, BET, or their combination can protect boar sperm from HP-induced damage by modulating the sphingolipid signaling pathway and alleviating alterations in PC and AcCa levels.

## 4. Discussion

In modern pig farming, the demand for reproductive efficiency is continuously increasing, and artificial insemination (AI) is a core component. Although technologies for semen preservation and fertilization outcomes are already quite advanced, there has been little improvement in the time efficiency of AI. If HP-induced damage in novel bionic insemination catheters can be alleviated, this would not only enhance insemination efficiency and reduce time costs but also ensure AI effectiveness, which has important practical significance for modern pig production. TMAO can alleviate cellular damage induced by HP by stabilizing cell membrane structures, reducing oxidative stress, protecting mitochondria, and regulating osmotic balance [17,18,19]. BET, on the other hand, can protect spermatozoa through osmotic protection, antioxidation, membrane stabilization, and mitochondrial protection. To date, studies on the role of TMAO in mitigating HP-induced sperm damage are limited. Previous research has shown that dietary supplementation with BET can increase sperm production in boars during summer and that adding BET to semen can reduce quality losses caused by storage and transportation [20,21]. Furthermore, supplementing rabbit diets with BET was found to reduce declines in motility, acrosome integrity, and plasma membrane integrity caused by heat stress [22]. However, no studies to date have focused on its role in alleviating HP-induced boar sperm damage.

Computer-assisted sperm analysis is crucial in assessing boar sperm quality under HP stress, as it rapidly and objectively evaluates sperm motility parameters. These parameters also provide insights into functional changes in sperm after stress exposure. In this study, results showed that HP stress significantly reduced sperm motility parameters, indicating a strong negative impact on sperm viability and functionality, which was further confirmed by viability assays. At 0 h post-loading, sperm viability decreased immediately after being introduced into the novel bionic insemination catheter, suggesting that the loading process itself induced partial sperm damage. Supplementation with 8 mmol/L TMAO or 20 mmol/L BET mitigated the decline in motility parameters during room-temperature storage to a certain extent. HP stress is thought to compress the sperm plasma membrane lipid bilayer, increasing its rigidity and reducing fluidity, thereby impairing capacitation and hindering the acrosome reaction [23,24]. Thus, the plasma membrane and acrosome are critical indicators for assessing sperm function. In this study, HP stress significantly decreased sperm plasma membrane integrity, while supplementation with TMAO, BET, or their combination effectively preserved membrane integrity. However, no significant differences were observed in acrosome integrity among the treatment groups under HP stress, suggesting that 105 kPa HP was insufficient to cause acrosomal damage.

Mitochondrial function in sperm is closely associated with energy production, redox homeostasis, and apoptotic pathways, and alterations in these functions can ultimately lead to a decline in sperm quality [25]. The present study demonstrates that supplementation with TMAO, BET, or their combination effectively mitigates HP-induced mitochondrial damage in boar sperm, thereby maintaining sperm quality, which is consistent with previously reported trends. Kadlec et al. [26] reported that elevated NO concentrations are associated with reduced semen quality. MDA, a product of lipid peroxidation, serves as an indicator of oxidative damage in sperm. In the present study, TMAO, BET, and their combination effectively reduced ROS accumulation, though not to the levels observed in the fresh group. They also significantly decreased MDA and NO contents, with T-AOC effectively maintained only in the BET group. OS can lead to decreased sperm motility, impaired membrane and DNA integrity, and increased lipid peroxidation [27]. Excessive DFI may compromise fertility; thus, reducing OS and optimizing semen preservation conditions, such as temperature and pressure, can help maintain DNA integrity [28,29]. Similarly, in this study, supplementation with TMAO, BET, or their combination effectively reduced DNA damage, thereby protecting sperm from HP-induced stress and oxidative damage.

Lipidomics is an omics approach that systematically investigates the species, structures, contents, and dynamic changes of lipids within cells, and it represents an important branch of metabolomics. Using high-resolution mass spectrometry techniques such as LC-MS and GC-MS, lipidomics enables comprehensive qualitative and quantitative profiling of cellular lipids, thereby facilitating the analysis of alterations in lipid metabolism [30]. Lipids play essential roles in biological systems, including energy storage, structural components of cell membranes, and intermediates of metabolism and signaling pathways. Lipid metabolism is highly complex, and its dysregulation is closely associated with the pathogenesis of various common diseases [31]. In sperm physiology, lipids are equally critical, not only influencing membrane fluidity and integrity but also participating in capacitation, membrane fusion during fertilization, the acrosome reaction, and preservation during cryostorage [32,33]. Currently, lipidomic studies addressing the capacity of TMAO or BET to alleviate HP-induced sperm damage remain scarce. Therefore, we employed LC-MS to investigate the regulatory effects of TMAO or BET on lipid metabolism in boar sperm under HP stress. The aim was to identify key lipid molecules contributing to sperm resistance to HP, optimize preservation strategies, and improve biological function, thereby providing scientific evidence and data to elucidate the mechanisms of TMAO, BET, or their combination, as well as HP-induced damage during liquid storage of boar sperm.

In this study, 286 differential lipids were identified by comparison between the pressurized control group and the fresh group, which may represent the key factors underlying alterations in lipid metabolism under HP stress. KEGG pathway enrichment revealed that HP primarily affected the sphingolipid signaling pathway, with significant decreases observed in Cer, DG, and SM. Additional enriched pathways included glycerophospholipid metabolism, adipocytokine signaling, insulin resistance, and choline metabolism in cancer. These findings are consistent with earlier experimental results, which demonstrated that HP impairs sperm plasma membrane integrity and fluidity, induces subsequent oxidative stress, and ultimately reduces sperm motility parameters. KEGG enrichment further indicated that HP mainly influences signaling pathways associated with membrane structure, apoptosis, and capacitation. Our experimental results confirmed that TMAO, BET, and TMAO + BET exert protective effects against HP-induced sperm damage, although the underlying mechanisms remained unclear. Lipidomic profiling revealed that compared with the pressurized control, the TMAO group contained 49 differential lipids, the BET group contained 262, and the TMAO + BET group contained 269. These results indicate that TMAO, BET, and their combination all influenced lipid activity in boar sperm following HP stress. The sphingolipid signaling pathway was enriched in the TMAO group, but only weakly, with Cer as the primary differential lipid. This suggests that the protective mechanism of TMAO may not rely on extensive regulation of sphingolipid signaling but instead acts through improving membrane lipid fluidity, reducing oxidative stress, or maintaining structural integrity of the plasma membrane. By contrast, the sphingolipid signaling pathway in the BET and TMAO + BET groups was mainly enriched in DG and Cer, suggesting that BET regulates sperm membrane lipid homeostasis, thereby contributing to resistance against HP-induced membrane damage.

The sphingolipid signaling pathway plays an essential role in cellular physiology, particularly in maintaining membrane structure, mediating signal transduction, and regulating apoptosis [34]. Cer is a central molecule in this pathway, regulating membrane fluidity and acting as a signaling mediator of apoptosis [35,36]. Under HP stress, alterations in Cer metabolism may trigger changes in sperm membrane structure, ultimately affecting sperm motility and viability. In this study, we found that Cer content in sperm was significantly reduced in the pressurized control group, which contrasts with the classical model in which oxidative stress induces Cer accumulation. This discrepancy suggests that sperm under HP stress may activate alternative lipid metabolic routes, such as conversion into ceramide-1-phosphate or sphingosine-1-phosphate, to reduce Cer-mediated apoptotic signaling. Nevertheless, such metabolic shifts may also impair membrane fluidity and structural stability, thereby influencing capacitation and fertilization competence. The effect of HP on sperm sphingolipid metabolism likely involves a more complex regulatory mechanism that warrants further investigation, particularly in connection with membrane dynamics, oxidative stress, and capacitation-related signaling pathways. Serafini et al. [37] reported that Cer not only serves as a key mediator in stress signaling but also represents an important biomarker for capacitation in human sperm. Changes in SM may influence sperm membrane stability and fertilizing ability [38]. DG, a critical intermediate in the phosphatidylinositol signaling pathway, is implicated in the regulation of capacitation and the acrosome reaction [39]. Chen et al. [40] observed that DG levels were reduced in the sperm of asthenozoospermic patients compared to normozoospermic controls. Significant alterations in AcCa under HP stress may impair mitochondrial function, oxidative balance, and membrane lipid composition, thereby compromising sperm viability and fertilizing capacity [41]. PC, the predominant phospholipid in the sperm membrane, is essential for maintaining structural integrity, fluidity, and stability, making its validation particularly important. PC has been proposed as a biomarker of sperm cryotolerance, potentially protecting sperm by preventing mechanical damage to the membrane and participating in lipid metabolism [32]. Garcia et al. [42] reported that specific fatty acid compositions were correlated with sperm membrane integrity, caspase activity, and lipid peroxidation, both positively and negatively. Tavilani et al. [43] demonstrated that changes in PC levels were associated with sperm quality in asthenozoospermic patients. Similarly, Lucio et al. [44] found that sperm quality in dogs varied considerably depending on membrane lipid composition, with PC identified as a lipid marker of sperm motility. ELISA kits were used to measure the levels of DG, PC, SM, Cer, and AcCa to validate the lipidomic findings. The validation results were consistent with the lipidomic analysis, supporting the hypothesis that BET and TMAO + BET protect sperm against HP stress by modulating differential lipid metabolites within the sphingolipid signaling pathway, thereby maintaining membrane homeostasis and fluidity. When TMAO was added alone, the enrichment of the sphingolipid signaling pathway was relatively weak. Compared with TMAO, BET exhibited a more pronounced protective effect in the present system, likely due to its dual role in participating in methyl metabolism to promote membrane phospholipid remodeling and enhancing cellular antioxidant capacity, thereby providing more comprehensive protection in maintaining membrane integrity and sperm motility. In contrast, the primary action of TMAO appears to be related to protein/solvent layer stabilization, resulting in a comparatively limited effect on lipid metabolism. Future studies are needed to further verify these mechanisms through assessments of enzyme activity, metabolic flux, and membrane biophysical properties. In summary, lipidomics was employed in this study to investigate the impact of HP on sperm lipid metabolism, and sphingolipid signaling and glycerophospholipid metabolism were identified as the key affected pathways. These changes were accompanied by oxidative stress and alterations in the membrane lipid environment. However, the effects of HP on sperm represent a complex physiological process, involving multiple interconnected mechanisms, including membrane stability, mitochondrial function, energy metabolism, and signaling regulation. Although potential mechanisms have been uncovered in this study, further investigations are needed to clarify the precise molecular regulation and physiological implications to achieve a comprehensive understanding of sperm adaptation and response to HP stress.

## 5. Conclusions

The addition of 8 mmol/L TMAO, 20 mmol/L BET, or their combination was able to maintain the morphological parameters, viability, and motility of boar sperm during liquid storage after exposure to 105 kPa HP stress, while reducing the impact of OS-related indicators and enhancing antioxidative capacity. This protective effect was achieved by maintaining lipid homeostasis, thereby alleviating HP-induced damage to sperm.

## Figures and Tables

**Table 1:** Effects of different concentrations of TMAO or BET on the motility of boar sperm under HP stress.

Group	Semen HP	0 h	48 h	96 h	144 h
Fresh group (0 mmol/L)	0 kPa	95.83 ± 1.63 *	94.25 ± 2.51 *	94.27 ± 2.20 *	94.14 ± 1.56 *
Control group (0 mmol/L)	105 kPa	93.53 ± 1.18 ^#^	89.88 ± 1.97 ^#^	89.23 ± 2.58 ^#^	84.11 ± 4.39 ^#^
1 mmol/L TMAO	105 kPa	93.49 ± 1.16 ^#^	92.00 ± 1.36 *^,#^	91.42 ± 2.46 *^,#^	91.36 ± 1.35 *^,#^
2 mmol/L TMAO	105 kPa	93.67 ± 1.58 ^#^	92.67 ± 1.78 *	91.55 ± 1.80 *^,#^	91.40 ± 2.02 *^,#^
4 mmol/L TMAO	105 kPa	93.73 ± 1.91 ^#^	92.99 ± 1.95 *	92.03 ± 1.08 *^,#^	91.62 ± 2.19 *^,#^
8 mmol/L TMAO	105 kPa	93.93 ± 2.25 ^#^	93.26 ± 1.85 *	92.94 ± 1.22 *	92.83 ± 2.23 *
16 mmol/L TMAO	105 kPa	93.84 ± 2.17 ^#^	91.91 ± 1.77 *^,#^	89.65 ± 2.83 ^#^	90.05 ± 1.68 *^,#^
5 mmol/L BET	105 kPa	93.55 ± 1.22 ^#^	91.36 ± 2.27 ^#^	89.28 ± 2.61 ^#^	87.08 ± 3.08 *^,#^
10 mmol/L BET	105 kPa	93.63 ± 2.24 ^#^	92.18 ± 2.78 *^,#^	91.07 ± 1.55 ^#^	91.12 ± 1.38 *^,#^
20 mmol/L BET	105 kPa	93.89 ± 1.87 ^#^	93.51 ± 1.51 *	92.08 ± 2.27 *^,#^	91.44 ± 1.35 *^,#^
40 mmol/L BET	105 kPa	93.85 ± 1.67 ^#^	91.08 ± 2.15 ^#^	87.68 ± 2.37 ^#^	87.36 ± 1.15 *^,#^
80 mmol/L BET	105 kPa	92.90 ± 0.57 ^#^	86.28 ± 3.82 *^,#^	85.48 ± 5.09 *^,#^	83.68 ± 3.31 ^#^

Data are presented as mean ± SD (*n* = 3). Symbols indicate statistical significance: ^#^ compared with the fresh control group, * compared with the HP control group, *p* < 0.05; no symbol indicates *p* > 0.05.

**Table 2:** Effects of TMAO or BET on sperm viability, plasma membrane integrity, acrosome integrity, MMP, and DFI of boar spermatozoa under HP stress during room-temperature storage.

Item	Fresh Group	Control Group	T Group	B Group	H Group
Plasma membrane integrity, %	70.49 ± 1.42 *	44.05 ± 4.11 ^#^	61.45 ± 1.78 *^,#^	60.84 ± 3.84 *^,#^	58.76 ± 2.52 *^,#^
Acrosome integrity, %	95.93 ± 1.27	96.13 ± 1.57	96.36 ± 1.31	96.57 ± 1.24	95.88 ± 0.81
Sperm viability, %	91.95 ± 2.65 *	84.14 ± 1.75 ^#^	90.36 ± 1.96 *	90.27 ± 1.57 *	89.69 ± 1.27 *^,#^
DFI, %	17.21 ± 2.31 *	21.98 ± 1.70 ^#^	18.73 ± 1.95 *	17.69 ± 0.71 *	18.17 ± 1.39 *
MMP	4.47 ± 0.48 *	3.29 ± 0.19 ^#^	4.19 ± 0.59 *	4.34 ± 0.42 *	4.37 ± 0.99 *

Data are presented as mean ± SD (*n* = 3). Symbols indicate statistical significance: ^#^ compared with the fresh control group, * compared with the HP control group, *p* < 0.05; no symbol indicates *p* > 0.05.

**Table 3:** Effects of TMAO or BET on ROS, T-AOC, NO, and MDA levels in boar sperm under HP stress during liquid storage at ambient temperature.

Item	Fresh Group	Control Group	T Group	B Group	H Group
ROS	1.00 ± 0.07 *	1.25 ± 0.06 ^#^	1.14 ± 0.05 *^,#^	1.12 ± 0.06 *^,#^	1.12 ± 0.04 *^,#^
T-AOC, mmol/L	2.36 ± 0.11 *	2.13 ± 0.06 ^#^	2.22 ± 0.07 ^#^	2.32 ± 0.07 *	2.17 ± 0.09 ^#^
NO, μmol/L	2.1 ± 0.64 *	3.55 ± 0.31 ^#^	2.36 ± 0.22 *	2.59 ± 0.3 *	2.42 ± 0.68 *
MDA, nmol/mg prot	2.86 ± 0.46 *	4.84 ± 0.5 ^#^	3.31 ± 0.47 *	3.72 ± 0.57 *	3.54 ± 0.41 *

Data are presented as mean ± SD (*n* = 3). Symbols indicate statistical significance: ^#^ compared with the fresh control group, * compared with the HP control group, *p* < 0.05; no symbol indicates *p* > 0.05.

## Data Availability

The data presented in this study are available on request from the corresponding author.

## Supplementary Materials

The following supporting information can be downloaded at: https://www.mdpi.com/article/10.3390/life15101606/s1, Table S1. Effects of different concentrations of TMAO or BET on kinematic parameters of boar sperm under HP stress; Figure S1. Representative images of boar sperm plasma membrane integrity, acrosome integrity, and viability; Figure S2. Representative images of boar sperm mitochondrial membrane potential (MMP) staining; Figure S3. Representative images of boar sperm DNA fragmentation index (DFI) staining; Figure S4. Representative fluorescence staining images of ROS in boar sperm; Figure S5. OPLS-DA score plot (left) and permutation test plot (right) of boar sperm stored at room temperature under HP stress (T vs. C, B vs. C, H vs. C); Figure S6. KEGG pathway enrichment analysis bubble plot of boar sperm stored at room temperature under HP stress (T vs. C, B vs. C, H vs. C).

## Abbreviations

The following abbreviations are used in this manuscript:

TMAO	Trimethylamine N-oxide
BET	Betaine
HP	Hydrostatic pressure
OS	Oxidative stress
ROS	Reactive oxygen species
TM	Total motility
VCL	Curvilinear velocity
VAP	Average path velocity
VSL	Straight-line velocity
MMP	Mitochondrial membrane potential
DFI	DNA fragmentation index
MDA	Malondialdehyde
NO	Nitric oxide
T-AOC	Total antioxidative capacity
OD	Optical density
QC	Quality control
