# Supplementary material for: Supplementation of Trimethylamine N-Oxide or Betaine in Semen Improves Quality of Boar Spermatozoa Stored at 17 °C Following Hydrostatic Pressure Stress
Source: Life (Basel). 2025 Oct 15;15(10):1606. doi: 10.3390/life15101606 (PMC12565095; doi:10.3390/life15101606)
Supplement: Supplementary file 1 [file life-15-01606-s001.zip › life-3880634-supplementary.pptx]

## Slide 1
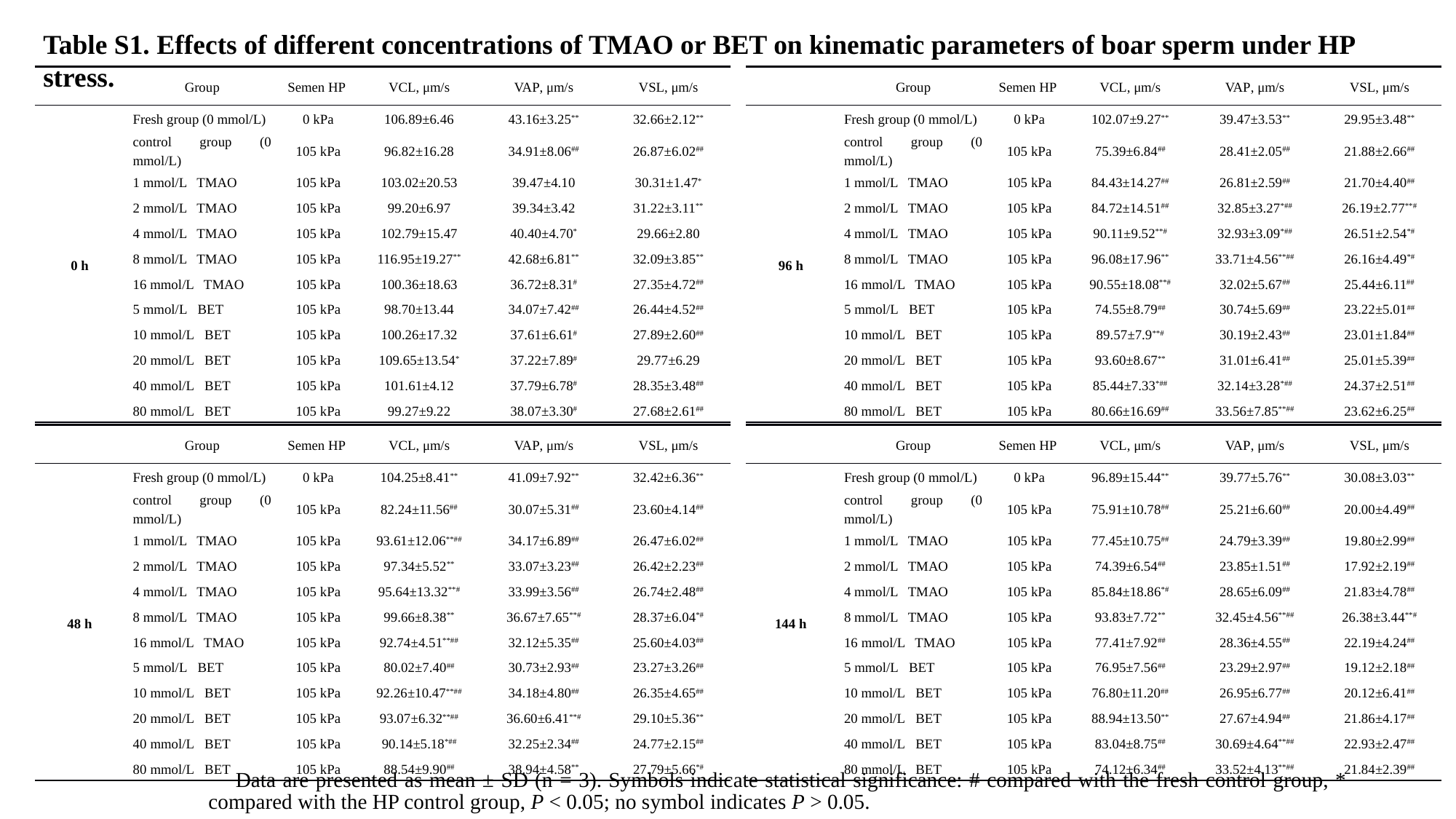

Table S1. Effects of different concentrations of TMAO or BET on kinematic parameters of boar sperm under HP stress.
| | Group | Semen HP | VCL, μm/s | VAP, μm/s | VSL, μm/s |
| --- | --- | --- | --- | --- | --- |
| 0 h | Fresh group (0 mmol/L) | 0 kPa | 106.89±6.46 | 43.16±3.25\*\* | 32.66±2.12\*\* |
| | control group (0 mmol/L) | 105 kPa | 96.82±16.28 | 34.91±8.06## | 26.87±6.02## |
| | 1 mmol/L TMAO | 105 kPa | 103.02±20.53 | 39.47±4.10 | 30.31±1.47\* |
| | 2 mmol/L TMAO | 105 kPa | 99.20±6.97 | 39.34±3.42 | 31.22±3.11\*\* |
| | 4 mmol/L TMAO | 105 kPa | 102.79±15.47 | 40.40±4.70\* | 29.66±2.80 |
| | 8 mmol/L TMAO | 105 kPa | 116.95±19.27\*\* | 42.68±6.81\*\* | 32.09±3.85\*\* |
| | 16 mmol/L TMAO | 105 kPa | 100.36±18.63 | 36.72±8.31# | 27.35±4.72## |
| | 5 mmol/L  BET | 105 kPa | 98.70±13.44 | 34.07±7.42## | 26.44±4.52## |
| | 10 mmol/L  BET | 105 kPa | 100.26±17.32 | 37.61±6.61# | 27.89±2.60## |
| | 20 mmol/L  BET | 105 kPa | 109.65±13.54\* | 37.22±7.89# | 29.77±6.29 |
| | 40 mmol/L  BET | 105 kPa | 101.61±4.12 | 37.79±6.78# | 28.35±3.48## |
| | 80 mmol/L  BET | 105 kPa | 99.27±9.22 | 38.07±3.30# | 27.68±2.61## |
| | Group | Semen HP | VCL, μm/s | VAP, μm/s | VSL, μm/s |
| --- | --- | --- | --- | --- | --- |
| 96 h | Fresh group (0 mmol/L) | 0 kPa | 102.07±9.27\*\* | 39.47±3.53\*\* | 29.95±3.48\*\* |
| | control group (0 mmol/L) | 105 kPa | 75.39±6.84## | 28.41±2.05## | 21.88±2.66## |
| | 1 mmol/L TMAO | 105 kPa | 84.43±14.27## | 26.81±2.59## | 21.70±4.40## |
| | 2 mmol/L TMAO | 105 kPa | 84.72±14.51## | 32.85±3.27\*## | 26.19±2.77\*\*# |
| | 4 mmol/L TMAO | 105 kPa | 90.11±9.52\*\*# | 32.93±3.09\*## | 26.51±2.54\*# |
| | 8 mmol/L TMAO | 105 kPa | 96.08±17.96\*\* | 33.71±4.56\*\*## | 26.16±4.49\*# |
| | 16 mmol/L TMAO | 105 kPa | 90.55±18.08\*\*# | 32.02±5.67## | 25.44±6.11## |
| | 5 mmol/L  BET | 105 kPa | 74.55±8.79## | 30.74±5.69## | 23.22±5.01## |
| | 10 mmol/L  BET | 105 kPa | 89.57±7.9\*\*# | 30.19±2.43## | 23.01±1.84## |
| | 20 mmol/L  BET | 105 kPa | 93.60±8.67\*\* | 31.01±6.41## | 25.01±5.39## |
| | 40 mmol/L  BET | 105 kPa | 85.44±7.33\*## | 32.14±3.28\*## | 24.37±2.51## |
| | 80 mmol/L  BET | 105 kPa | 80.66±16.69## | 33.56±7.85\*\*## | 23.62±6.25## |
| | Group | Semen HP | VCL, μm/s | VAP, μm/s | VSL, μm/s |
| --- | --- | --- | --- | --- | --- |
| 48 h | Fresh group (0 mmol/L) | 0 kPa | 104.25±8.41\*\* | 41.09±7.92\*\* | 32.42±6.36\*\* |
| | control group (0 mmol/L) | 105 kPa | 82.24±11.56## | 30.07±5.31## | 23.60±4.14## |
| | 1 mmol/L TMAO | 105 kPa | 93.61±12.06\*\*## | 34.17±6.89## | 26.47±6.02## |
| | 2 mmol/L TMAO | 105 kPa | 97.34±5.52\*\* | 33.07±3.23## | 26.42±2.23## |
| | 4 mmol/L TMAO | 105 kPa | 95.64±13.32\*\*# | 33.99±3.56## | 26.74±2.48## |
| | 8 mmol/L TMAO | 105 kPa | 99.66±8.38\*\* | 36.67±7.65\*\*# | 28.37±6.04\*# |
| | 16 mmol/L TMAO | 105 kPa | 92.74±4.51\*\*## | 32.12±5.35## | 25.60±4.03## |
| | 5 mmol/L  BET | 105 kPa | 80.02±7.40## | 30.73±2.93## | 23.27±3.26## |
| | 10 mmol/L  BET | 105 kPa | 92.26±10.47\*\*## | 34.18±4.80## | 26.35±4.65## |
| | 20 mmol/L  BET | 105 kPa | 93.07±6.32\*\*## | 36.60±6.41\*\*# | 29.10±5.36\*\* |
| | 40 mmol/L  BET | 105 kPa | 90.14±5.18\*## | 32.25±2.34## | 24.77±2.15## |
| | 80 mmol/L  BET | 105 kPa | 88.54±9.90## | 38.94±4.58\*\* | 27.79±5.66\*# |
| | Group | Semen HP | VCL, μm/s | VAP, μm/s | VSL, μm/s |
| --- | --- | --- | --- | --- | --- |
| 144 h | Fresh group (0 mmol/L) | 0 kPa | 96.89±15.44\*\* | 39.77±5.76\*\* | 30.08±3.03\*\* |
| | control group (0 mmol/L) | 105 kPa | 75.91±10.78## | 25.21±6.60## | 20.00±4.49## |
| | 1 mmol/L TMAO | 105 kPa | 77.45±10.75## | 24.79±3.39## | 19.80±2.99## |
| | 2 mmol/L TMAO | 105 kPa | 74.39±6.54## | 23.85±1.51## | 17.92±2.19## |
| | 4 mmol/L TMAO | 105 kPa | 85.84±18.86\*# | 28.65±6.09## | 21.83±4.78## |
| | 8 mmol/L TMAO | 105 kPa | 93.83±7.72\*\* | 32.45±4.56\*\*## | 26.38±3.44\*\*# |
| | 16 mmol/L TMAO | 105 kPa | 77.41±7.92## | 28.36±4.55## | 22.19±4.24## |
| | 5 mmol/L  BET | 105 kPa | 76.95±7.56## | 23.29±2.97## | 19.12±2.18## |
| | 10 mmol/L  BET | 105 kPa | 76.80±11.20## | 26.95±6.77## | 20.12±6.41## |
| | 20 mmol/L  BET | 105 kPa | 88.94±13.50\*\* | 27.67±4.94## | 21.86±4.17## |
| | 40 mmol/L  BET | 105 kPa | 83.04±8.75## | 30.69±4.64\*\*## | 22.93±2.47## |
| | 80 mmol/L  BET | 105 kPa | 74.12±6.34## | 33.52±4.13\*\*## | 21.84±2.39## |
Data are presented as mean ± SD (n = 3). Symbols indicate statistical significance: # compared with the fresh control group, * compared with the HP control group, P < 0.05; no symbol indicates P > 0.05.

## Slide 2
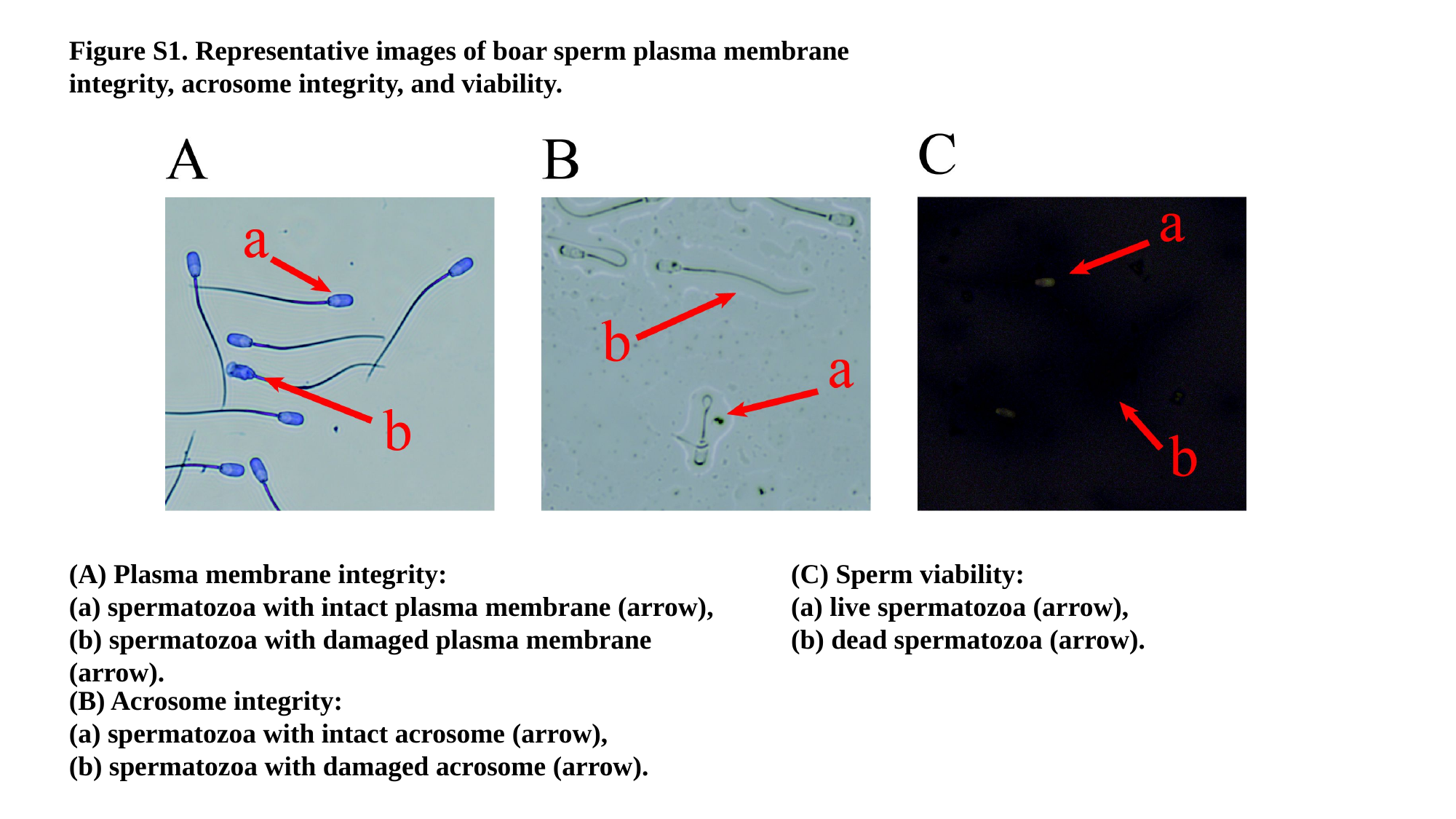

Figure S1. Representative images of boar sperm plasma membrane integrity, acrosome integrity, and viability.
(A) Plasma membrane integrity:
(a) spermatozoa with intact plasma membrane (arrow),
(b) spermatozoa with damaged plasma membrane (arrow).
(C) Sperm viability:
(a) live spermatozoa (arrow),
(b) dead spermatozoa (arrow).
(B) Acrosome integrity:
(a) spermatozoa with intact acrosome (arrow),
(b) spermatozoa with damaged acrosome (arrow).

## Slide 3
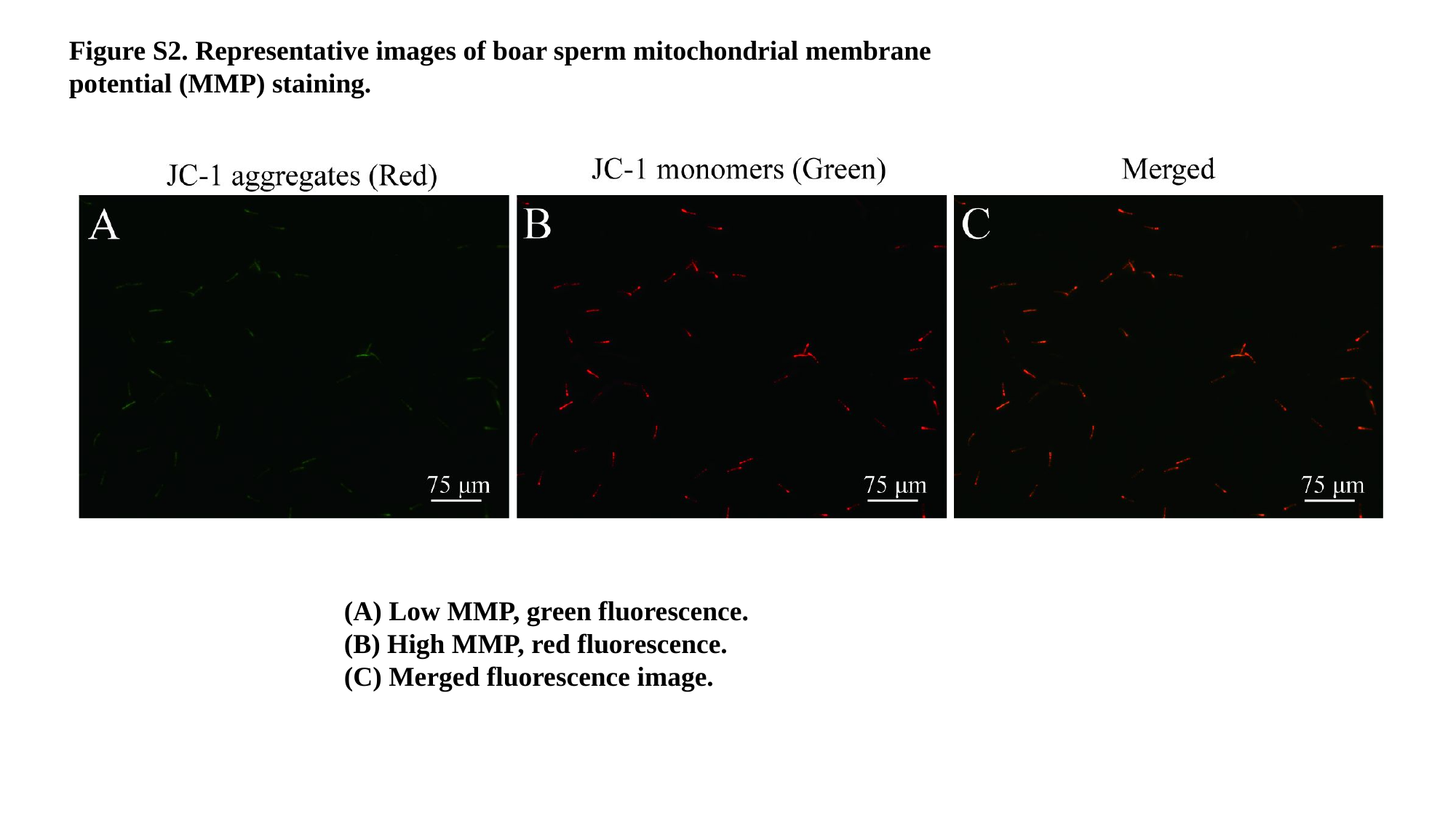

Figure S2. Representative images of boar sperm mitochondrial membrane potential (MMP) staining.
(A) Low MMP, green fluorescence.
(B) High MMP, red fluorescence.
(C) Merged fluorescence image.

## Slide 4
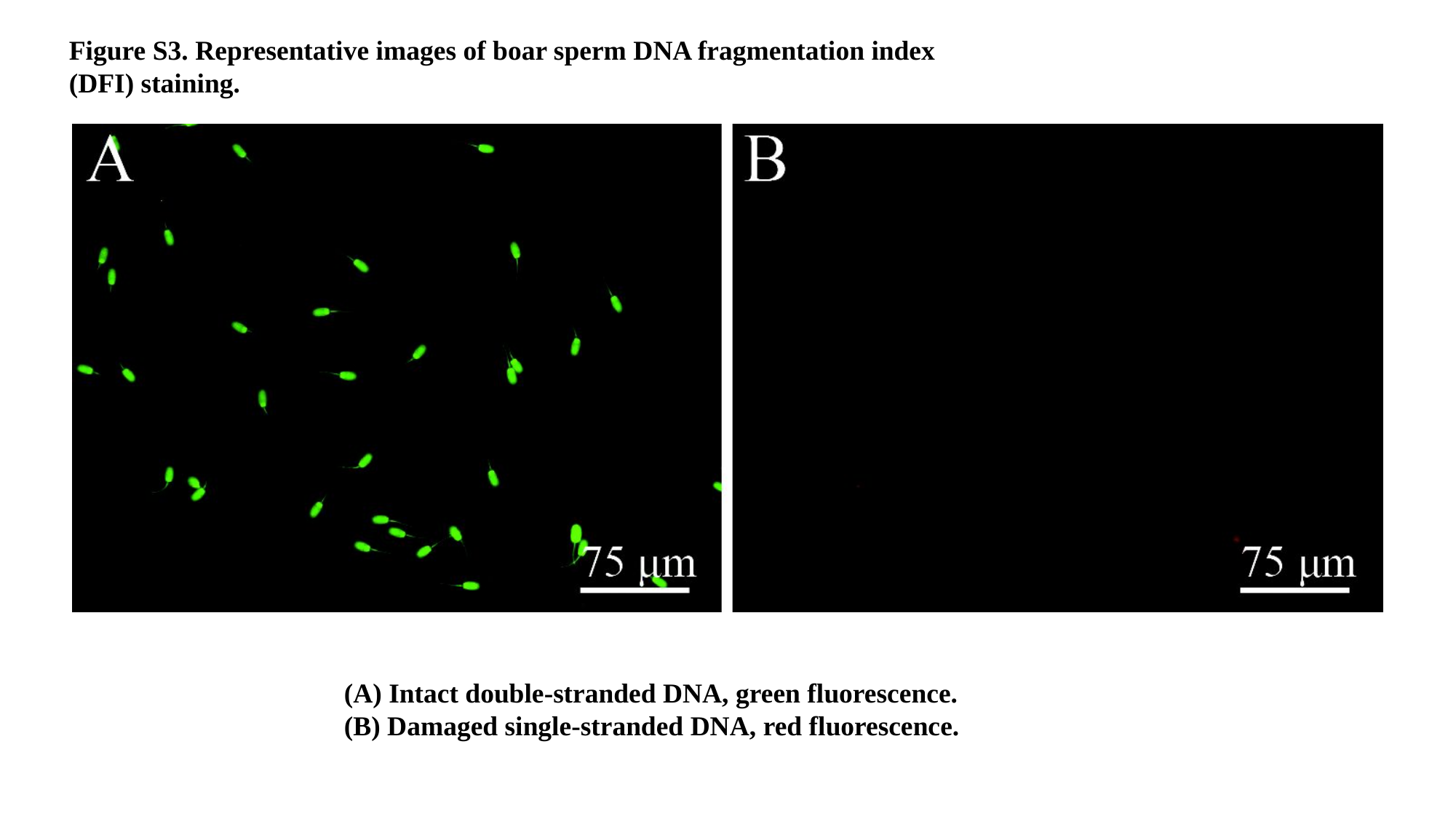

Figure S3. Representative images of boar sperm DNA fragmentation index (DFI) staining.
(A) Intact double-stranded DNA, green fluorescence.
(B) Damaged single-stranded DNA, red fluorescence.

## Slide 5
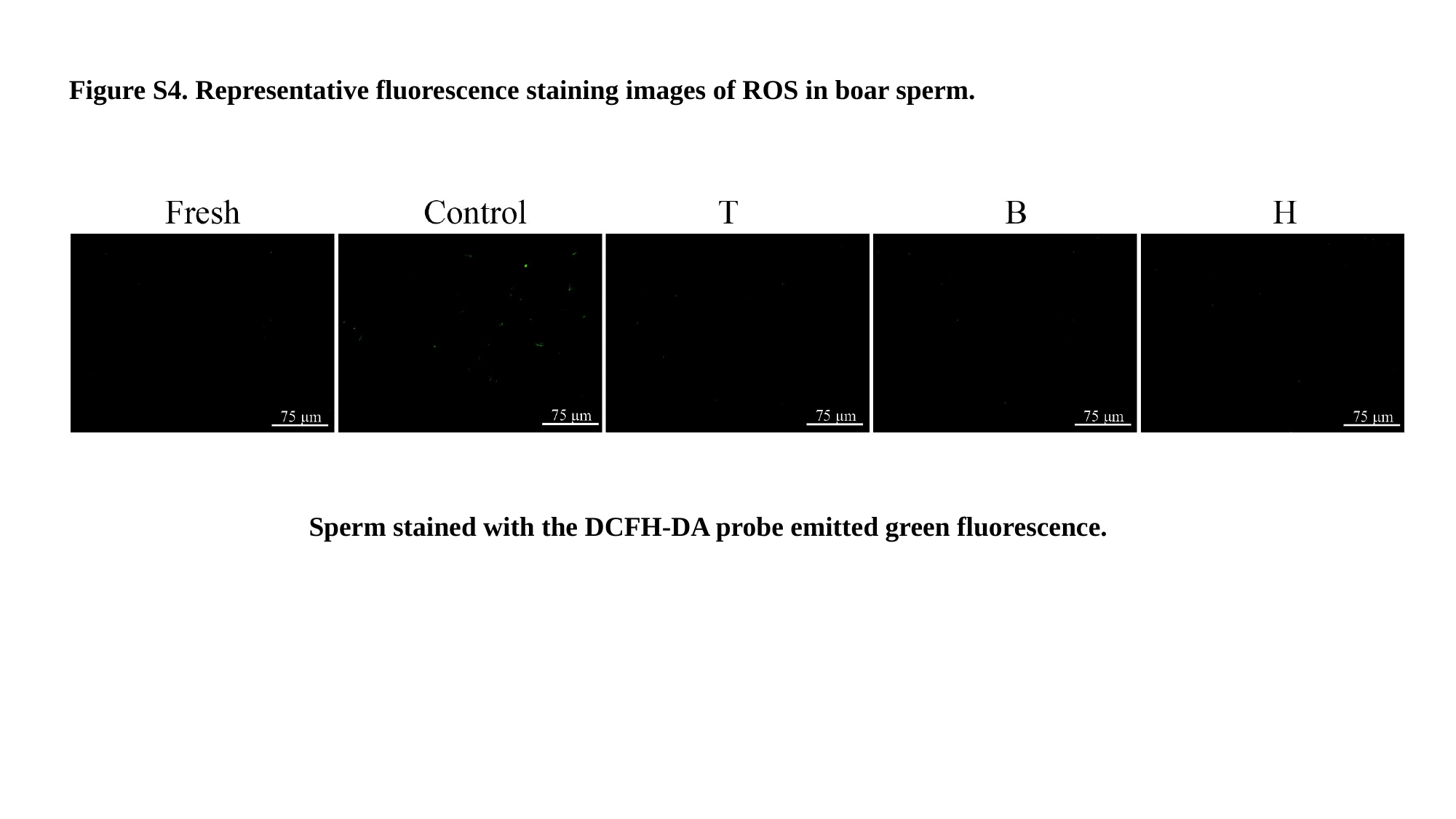

Figure S4. Representative fluorescence staining images of ROS in boar sperm.
Sperm stained with the DCFH-DA probe emitted green fluorescence.

## Slide 6
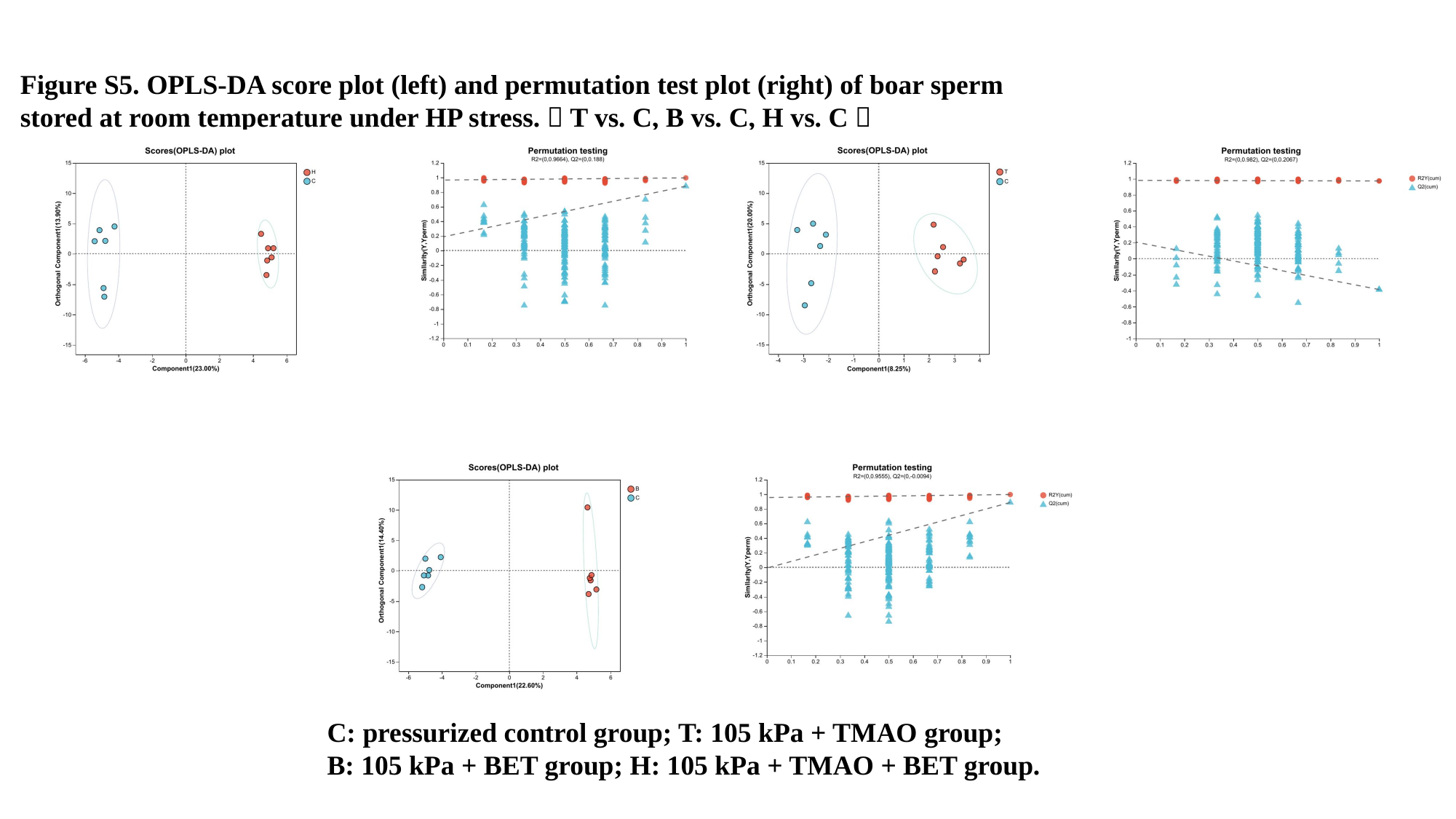

Figure S5. OPLS-DA score plot (left) and permutation test plot (right) of boar sperm stored at room temperature under HP stress.（T vs. C, B vs. C, H vs. C）
C: pressurized control group; T: 105 kPa + TMAO group;
B: 105 kPa + BET group; H: 105 kPa + TMAO + BET group.

## Slide 7
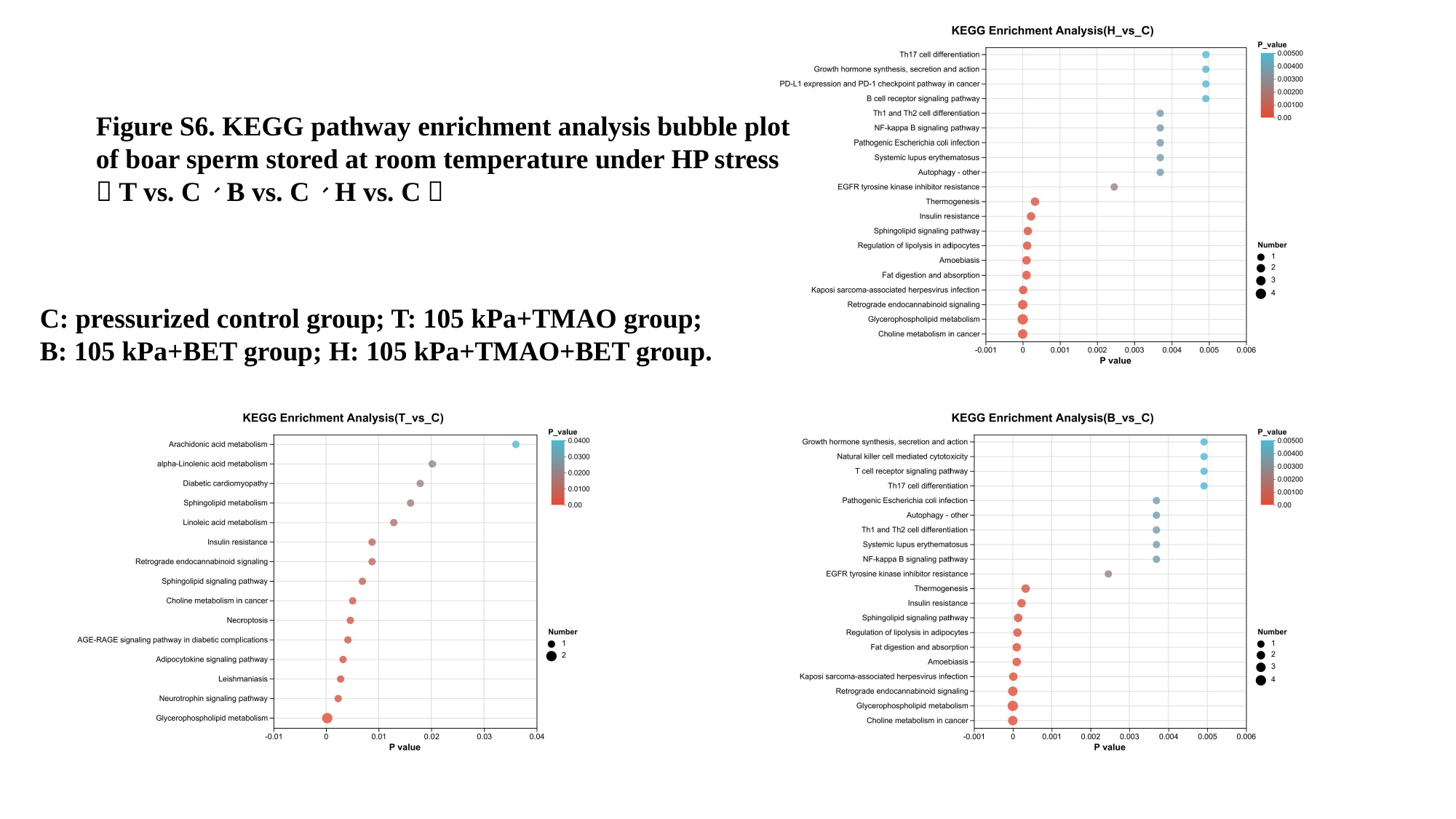

Figure S6. KEGG pathway enrichment analysis bubble plot of boar sperm stored at room temperature under HP stress
（T vs. C、B vs. C、H vs. C）
C: pressurized control group; T: 105 kPa+TMAO group;
B: 105 kPa+BET group; H: 105 kPa+TMAO+BET group.
